# Efficacy and safety of grain moxibustion in hemiplegia: A systematic review and meta-analysis protocol

**DOI:** 10.1097/MD.0000000000015215

**Published:** 2019-04-26

**Authors:** Guoming Chen, Chuyao Huang, Yunyun Liu, Zhaoping Zhang, Xiangjun Qi, Peiyu Shi, Dan Li, Huiye Lv, Bin Zhang

**Affiliations:** a Guangzhou University of Chinese Medicine; b Department of acupuncture and moxibustion, First Affiliated Hospital of Guangzhou University of Chinese Medicine, Guangzhou, China.

**Keywords:** Grain moxibustion, hemiplegia, protocol, systematic review

## Abstract

**Background:**

The increasing morbidity of stroke brings enormous social and economic pressure to the countries. Hemiplegia is the common sequela of stroke patients, which affects their physical activities and daily life, and its optimal treatment is still an urgent problem. In Asian countries, moxibustion therapy is widely combined with rehabilitation in poststroke hemiplegia. Among them, grain moxibustion, due to its concentration on stimulating the acupoints and deep heat penetration, can promote neurorehabilitation after stroke. Therefore, based on the current literatures, the effectiveness and safety of grain moxibustion were systematically evaluated to provide possible alternative therapy for the rehabilitation of hemiplegia.

**Methods:**

Studies search for eligible randomized controlled trials (RCTs) that use grain moxibustion as the sole treatment for hemiplegia and their data extraction will be done by two researchers. Mean difference (MD) or relative risk (RR) with fixed or random effect model in terms of 95% confidence interval (CI) will be adopted for the data synthesis. To evaluate the risk of bias, the Cochrane's risk of bias assessment tool will be utilized. The sensitivity or subgroup analysis will also be conducted when meeting high heterogeneity (*I*^2^ > 50%).

**Results:**

This meta-analysis will provide an authentic synthesis of the grain moxibustion's effect for hemiplegia.

**Conclusion:**

The findings of the review offer updated evidence and identify whether grain moxibustion can be an effective treatment for hemiplegia.

**PROSPERO registration number:**

PROSPERO CRD 42018117765

## Abstract 2

Supplemental Digital Content is available in the text

## 1 Introduction

Stroke is an acute cerebrovascular event caused by brain ischemia, which has become the second leading cause of death.^[1,2]^ And 90% of poststroke patients have complications such as cognitive deficits and dyskinesia, which impose huge economic and social burden on the country.^[3]^ Hemiplegia is a common sequela of stroke, which restricts limb movement, and spastic hemiplegia has become a rehabilitation difficulty for 38% of poststroke patients.^[4,5]^ At present, botulinum neurotoxin injection therapy combined with rehabilitation has been applied for stroke patients to reduce the spasticity of limbs.^[6]^ However, as a bacterial endotoxin, botulinum toxin can cause allergic reactions. And other rehabilitation methods in hemiplegia have not found optimal treatment yet.^[7]^

In Asia, acupuncture and moxibustion are widely used in the rehabilitation of stroke patients with hemiplegia as the comprehensive therapy.^[8]^ As a percutaneous therapy, moxibustion can permeate the volatile components and heat into acupoints through burned mugwort (*Artemisia vulgaris*) floss.^[9,10]^ According to the theory of TCM, hemiplegia in stroke is mainly related to the imbalance of qi and blood. Moxibustion can warm up cold-phlegm and dispel wind by its drug action and warm effect, and further harmonize qi and blood to facilitate recovery from cerebral ischemic.^[11]^

Grain moxibustion, different from indirect moxibustion, has the advantages of gentle stimulation, concentrated position, and better long-term effect through directly acting on acupoints.^[12]^ Avoiding the pain of acupuncture and overdose inhalation of mugwort smoke, it not only improves the peripheral circulation but also affects the central cerebral cortex reflectively and reduces muscle tension.^[13]^ As there is no meta-analysis on grain moxibustion therapy for hemiplegia after stroke, the purpose of this paper is to assess the quality of current randomized controlled trials and further systematically evaluate the effectiveness and safety of grain moxibustion.

## 2 Methods

### 2.1 Inclusion criteria for study selection

#### 2.1.1 Types of studies

All the RCTs of grain moxibustion as the treatment for hemiplegia will be involved in the review and no language or publication status limitation will be imposed.

#### 2.1.2 Types of participants

Patients included will be clinically diagnosed with hemiplegia and there is no restriction on sex, age, race, or duration of the disease. Besides, studies that do not mention diagnostic criteria will be ruled out.

#### 2.1.3 Types of interventions

The sole therapy applied in the treatment group is grain moxibustion, with all types of point combination being acceptable.

#### 2.1.4 Types of outcome measures

##### 2.1.4.1 Primary outcomes

1. Modified Ashworth scale (MAS) score
2. fugl-meyer rating scale (FMA) score
3. Barthel index (BI) score, clinic spasticity index (CSI)

##### 2.1.4.2 Secondary outcomes

1. Clinic spasticity index (CSI)
2. Electromyography value
3. Quality of life
4. Adverse events

### 2.2 Search methods for the identification of studies

#### 2.2.1 Electronic searches

The data collection will be conducted systematically by two researchers through eight databases from their inception to the present date. The databases to be searched for eligible RCTs are as follows: PubMed, EMBASE, Cochrane Library, Web of Science, China National Knowledge Infrastructure (CNKI), Wanfang database, Technology Periodical database (VIP), and China Biology Medicine Database (CBM). The final retrieval strategy for PubMed will be ruled in conjunction with medical and uncontrolled terms following several preretrievals, and will be shown in Supplement 1. Similar strategies will be applied to other databases after adjustment.

#### 2.2.2 Other searches

Taking account of possible omission, not only the published studies in journals but also gray literatures will be retrieved, mainly through conference papers and references.

### 2.3 Data collection and analysis

#### 2.3.1 Selection of studies

**Figure 1:**
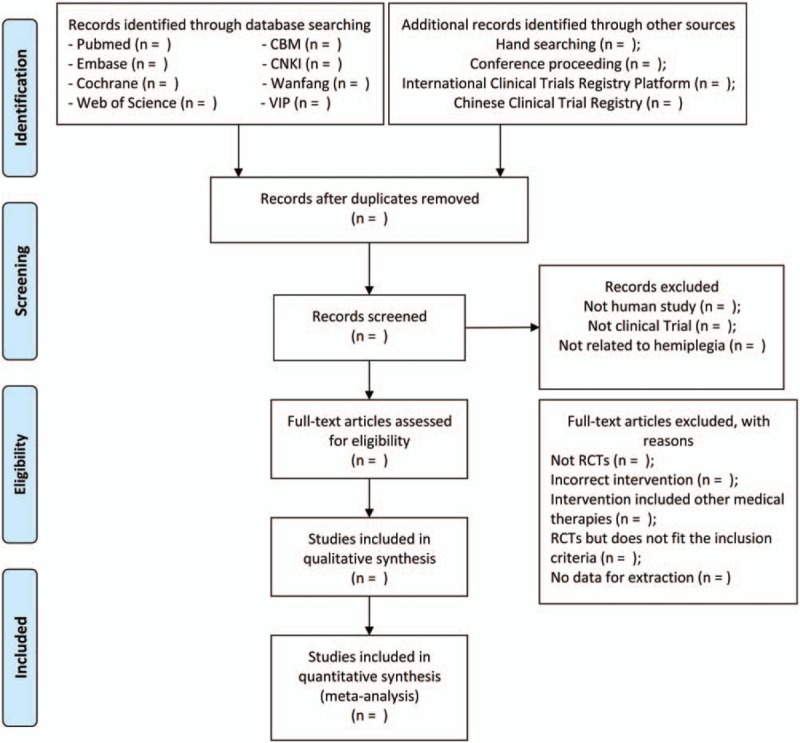
PRISMA flow chart of study selection process.

The selection of studies will be accomplished by two researches (ZZ and XQ) independently and be cross-checked. For literature management, EndnoteX9 will be utilized, with which the collected literatures will be imported and the duplicate ones will be deleted. Firstly, researchers will screen the titles and abstracts of the articles and exclude the distinctly ineligible ones. Afterward, the full text will be attentively inspected according to the inclusion and exclusion criteria previously set up. A third researcher will take part in the discussion and make the arbitration where there are discrepancies. The whole selection process will be presented in a PRISMA flow diagram (Fig. 1).

#### 2.3.2 Data extraction and management

Using a predefined data collection form, two researchers will complete the data extraction. Extracted data shall include but not be limited to the following items: title, first author, publication time, sample size, severity and duration of hemiplegia, age and gender of participants, outcomes, and adverse events. Inconsistencies will be resolved through discussion and consultation with a third researcher.

#### 2.3.3 Coping with the questionable data

When meeting reported data with ambiguity, contradiction, errors, or omission, the researcher will contact the first author for the clarified, correct, or missing data. Additionally, the potential impact of the questionable data will be discussed in the discussion part.

#### 2.3.4 Assessment of risk of bias in included studies

Two reviewers will separately appraise the risk of bias of the involved studies according to Cochrane Handbook for Systematic Reviews of Intervention. There will be three levels of evaluation results utilizing three scores of “L,’, “U,” and “H,” respectively, indicating low-risk, uncertain, and high-risk. In the appraisal, seven sectors will be assessed, including random sequence generation, allocation concealment, blinding of participants and personnel, blinding of outcome assessment, completeness of outcome data, selective reporting, and other sources of bias. Discussion with a third reviewer will be conducted to cope with potential divergence, and the corresponding author will be connected when needed.

#### 2.3.5 Measures of treatment effect

To appraise the treatment effect for continuous data, mean difference (MD) in terms of 95% confidence interval (CI) will be applied, just as relative risk (RR) for dichotomous data.

#### 2.3.6 Assessment of heterogeneity

On the basis of the data analysis, random effect or fixed effect models will be employed according to the heterogeneity given by *I*^2^ statistic value. To be concrete, a fixed effect model will be adopted if the heterogeneity is indicated as high (*I*^2^ < 50%); otherwise, a random effect model will be applied on the contrary.

#### 2.3.7 Assessment of reporting bias

The reporting bias will be visually indicated by funnel plots when studies are sufficient (at least 10 RCTs). If asymmetry is shown, Begg's and Egger's test will be completed and value of *P* > .05 will be interpreted as no substantial reporting bias. Since the asymmetry of the funnel plot is not a substitute to publication bias, the potential reasons for it will be discriminated with terms like small sample size, low methodological quality, or true heterogeneity.

#### 2.3.8 Data synthesis

The systematic review will be conducted with the use of RevMan 5.3. Taking account of the heterogeneity assessment, MD or RR with fixed or random effect model will be computed. Additionally, if heterogeneity is considered significant, the sensitivity or subgroup analysis will be generated to distinguish the source of it. When it comes to the situation that the data are insufficient for quantitative analysis, the review will only represent and summarize the evidence.

#### 2.3.9 Sensitivity analysis

As is mentioned above, sensitivity analysis will be done when the heterogeneity is greater than 50%. Concretely, the meta-analysis will be reconducted after the studies with low quality, small sample size, or older age are excluded to identify whether these factors influence the result.

#### 2.3.10 Subgroup analysis

Subgroup analysis will also be performed after substantial heterogeneity is observed to find out the reasons. Characteristics like outcome type, disease duration, study quality, race, and so forth will be the content of the subgroup analysis.

#### 2.3.11 Quality of evidence

To appraise the evidence quality more objectively, the reviewer will use the Grading of Recommendations Assessment, Development and Evaluation (GRADE), and complete the Summary of Findings table.

#### 2.3.12 Ethics and dissemination

Given that this protocol is for a systematic review, which involves no privacy data, ethical approval, and informed consent are needless. The results of this review will be disseminated widely through being submitted to peer-reviewed publications and conference presentations.

## 3 Discussion

Hemiplegia is common in stroke survivors, characterized by motor impairment of face and limbs of one side of the body. It may occur in the early stage of stroke, or worse yet, persist into the rest of the life of patients, thus seriously damaging the quality of life.^[7]^ The main objectives of rehabilitation in stroke patients with hemiplegia are therefore to stimulate the recovery of motor functions, and then maximize the prognosis of patients.

To date, quite a number of interventions for the hemiplegia rehabilitation have been developed over the past few decades, including robotics therapy, virtual reality, constraint-induced movement therapy, electrical stimulation, pharmacologic therapies, etc.^[7,14,15]^ However, their efficacy is still limited and there is remaining considerable confusion about the optimal treatment for hemiplegia because of its multifactorial etiology and complex underlying mechanisms.^[14,15]^

In recent years, clinical practices and some studies have also found that complementary and alternative medicine could offer great benefits to rehabilitation of hemiplegia, like acupuncture, cupping, and moxibustion.^[16–18]^ Grain moxibustion is a type of direct moxibustion, whose moxa cone is shaped into wheat grains size, widely used in rehabilitation of hemiplegia. Although its mechanism remains unclear, some studies have considered it as a promising intervention for its possible functions of reducing limb spasm, stimulating motor recovery and improving daily living activities.^[3,19,20]^

However, there is no available systematic review for the effectiveness and safety of grain moxibustion in rehabilitation of stroke patients with hemiplegia. We hope this systematic review can help comprehensively to evaluate its efficacy and safety, and contribute to the management of stroke patients with hemiplegia.

## Author contributions

Bin Zhang is the guarantor of the article and will be the arbitrator when meeting disagreements. All research members participated in developing the criteria and drafting the protocol for this systematic review. GC, CH, and YL established the search strategy. ZZ, XQ, and PS will independently accomplish the study selection, data extraction and assess the risk of bias. GC, DL, and HL will perform the data syntheses. The subsequent and final versions of the protocol are critically reviewed, modified, and authorized by all authors.

**Conceptualization:** Guoming Chen.

**Data curation:** Guoming Chen, Zhaoping Zhang, Xiangjun Qi, Peiyu Shi.

**Investigation:** Chuyao Huang, Yunyun Liu.

**Methodology:** Guoming Chen, Chuyao Huang, Yunyun Liu, Xiangjun Qi, Peiyu Shi.

**Project administration:** Guoming Chen, Chuyao Huang, Zhaoping Zhang, Bin Zhang.

**Supervision:** Guoming Chen, Bin Zhang.

**Validation:** Dan Li.

**Writing – original draft:** Guoming Chen, Chuyao Huang, Yunyun Liu, Zhaoping Zhang, Xiangjun Qi, Peiyu Shi, Dan Li, Huiye Lv.

**Writing – review & editing:** Guoming Chen, Bin Zhang.

Bin Zhang orcid: 0000-0002-5396-1441.

## Supplementary Material

Supplemental Digital Content
