# Supplementary material for: Efficacy and safety of grain moxibustion in hemiplegia: A systematic review and meta-analysis protocol
Source: Medicine (Baltimore). 2019 Apr 26;98(17):e15215. doi: 10.1097/MD.0000000000015215 (PMC6831348; doi:10.1097/MD.0000000000015215)
Supplement: Supplemental Digital Content [file medi-98-e15215-s001.doc]

**Supplement 1. Search strategy used in PubMed database**

#1 Hemiplegias OR Hemiplegia, Transient OR Hemiplegias, Transient OR Transient Hemiplegia OR Transient Hemiplegias OR Monoplegia OR Monoplegias OR Hemiplegia, Post-Ictal OR Hemiplegia, Post Ictal OR Hemiplegias, Post-Ictal OR Post-Ictal Hemiplegia OR Post-Ictal Hemiplegias OR Hemiplegia, Crossed OR Crossed Hemiplegia OR Crossed Hemiplegias OR Hemiplegias, Crossed OR Hemiplegia, Flaccid OR Flaccid Hemiplegia OR Flaccid Hemiplegias OR Hemiplegias, Flaccid OR Hemiplegia, Infantile OR Hemiplegias, Infantile OR Infantile Hemiplegia OR Infantile Hemiplegias OR Hemiplegia, Spastic OR Hemiplegias, Spastic OR Spastic Hemiplegia OR Spastic Hemiplegias

#2 Grain-moxibustion OR Grain moxibustion OR Moxibustion with seed-sized moxa cone OR Grain sized moxibustion OR moxibustion

#3 Randomized controlled trial OR clinical study OR Clin-ical Trial OR Controlled study OR Controlled Trial OR Random*Control* study OR random* Control* Trial

#4 #1 AND #2 AND #3
